# AI for Sustainable Recycling: Efficient Model Optimization for Waste Classification Systems

**DOI:** 10.3390/s25123807

**Published:** 2025-06-18

**Authors:** Oriol Chacón-Albero, Mario Campos-Mocholí, Cédric Marco-Detchart, Vicente Julian, Jaime Andrés Rincon, Vicent Botti

**Affiliations:** 1 Valencian Research Institute for Artificial Intelligence (VRAIN), Universitat Politècnica de València (UPV), Camino de Vera s/n, 46022 València, Spain; macammoc@upv.es (M.C.-M.); vjulian@upv.es (V.J.); vbotti@dsic.upv.es (V.B.); 2 Departamento de Estadística, Informática y Matemáticas, Universidad Pública de Navarra, Campus Arrosadia, 31006 Pamplona, Spain; cedric.marco@unavarra.es; 3 Valencian Graduate School and Research Network of Artificial Intelligence (VALGRAI), Universitat Politècnica de València (UPV), Camí de Vera s/n, 46022 València, Spain; 4 Departamento de Digitalización, Universidad de Burgos, 09006 Burgos, Spain; jarincon@ubu.es

**Keywords:** recycling, model quantization, image classification, aggregation functions

## Abstract

The increasing volume of global waste presents a critical environmental and societal challenge, demanding innovative solutions to support sustainable practices such as recycling. Advances in Computer Vision (CV) have enabled automated waste recognition systems that guide users in correctly sorting their waste, with state-of-the-art architectures achieving high accuracy. More recently, attention has shifted toward lightweight and efficient models suitable for mobile and edge deployment. These systems process data from integrated camera sensors in Internet of Things (IoT) devices, operating in real time to classify waste at the point of disposal, whether embedded in smart bins, mobile applications, or assistive tools for household use. In this work, we extend our previous research by improving both dataset diversity and model efficiency. We introduce an expanded dataset that includes an organic waste class and more heterogeneous images, and evaluate a range of quantized CNN models to reduce inference time and resource usage. Additionally, we explore ensemble strategies using aggregation functions to boost classification performance, and validate selected models on real embedded hardware and under simulated lighting variations. Our results support the development of robust, real-time recycling assistants for resource-constrained devices. We also propose architectural deployment scenarios for smart containers, and cloud-assisted solutions. By improving waste sorting accuracy, these systems can help reduce landfill use, support citizen engagement through real-time feedback, increase material recovery, support data-informed environmental decision making, and ease operational challenges for recycling facilities caused by misclassified materials. Ultimately, this contributes to circular economy objectives and advances the broader field of environmental intelligence.

## 1. Introduction

The problem of increasing global waste generation remains a fundamental challenge in the pursuit of sustainable development. Insufficient action in this area contributes to a wide range of environmental and social issues, including ocean plastic accumulation, health impacts through the food chain, nitrogen contamination, and greenhouse gas emissions, among several others. Maintaining current trends and incremental progress through existing approaches will fail to meaningfully ease ecological strain or enable the transformative restructuring needed to establish a circular economy [1].

In light of this escalating crisis, governments and organizations worldwide have adopted ambitious goals to encourage more sustainable practices, particularly in waste recycling. For example, the European Union has committed to achieving 60% of municipal waste prepared for reuse and recycling by 2030 [2]. Yet, despite these efforts, many countries still face significant barriers to achieving these targets. In Spain, where recycling rates stand at only 42.9% based on the latest registered data, still below the European average of 49.1% [3], the need for innovative, scalable solutions is particularly pressing.

One promising area for digital innovation lies in household recycling support systems that provide users with targeted information about recyclable items and proper disposal methods. The confusion surrounding waste sorting at the consumer level remains a significant barrier to improving recycling rates [4]. Different studies have shown that Computer Vision models offer considerable potential for addressing this challenge through automated waste recognition systems.

Early research efforts in 2016 [5] introduced one of the first benchmark datasets in this domain (the TrashNet Dataset) with six common waste categories, and explored the application of Convolutional Neural Networks (CNNs) and Support Vector Machines (SVMs) for classifying waste images. Building on this foundation and fueled by rapid advances in deep learning, more recent studies have achieved state-of-the-art performance using deep CNN architectures for waste classification tasks. For example, transfer learning techniques based on InceptionV3 achieved 95.33% in office waste classification [6]; an Xception model pre-trained on ImageNet surpassed both VGG-16 and ResNet-50 in multiclass classification, reaching an 87.92% accuracy in the TrashNet dataset [7]. A recent study [8] introduced the new Garbage Dataset, on which models from the EfficientNetV2 family outperformed MobileNet and ResNet variants. After an operational carbon emissions study, the EfficientNetV2-S model attained the best balance between performance (96.07% accuracy) and environmental impact.

In addition to household recycling, machine learning classification techniques are increasingly being applied across industrial waste streams. In textile recycling, automated systems using attention-based models have been deployed to sort post-consumer garments under complex visual conditions [9]. A broader systems-level perspective [10] highlights how innovations like smart tagging, machine vision, and hyperspectral imaging are helping automate fiber separation and disassembly processes. Similarly, deep learning has been employed in high-speed sorting lines for metal scrap to detect prohibited elements in real time [11], and in coal–gangue separation, where precise instance boundary detection enables robotic grasping of heterogeneous waste [12]. These applications often integrate sensors, machine vision, and robotic systems to optimize safety and efficiency in industrial contexts, offering useful parallels for consumer-facing smart waste systems.

In recent years, there has been increasing interest in developing integrated waste management systems that combine deep learning models with Internet of Things (IoT) infrastructure, including smart containers, mobile applications, cloud-based analytics, and sensor networks that monitor visual input, bin fill levels, gas emissions, weight, etc. These systems aim not only to classify waste at the source but also to support broader urban waste management through real-time monitoring, data analytics, and citizen engagement. For example, Li et al. [13] propose a smart trash can architecture that combines multiple detection and classification models for automatic waste separation while transmitting relevant data to the community management department. Although effective, their approach remains computationally demanding, emphasizing the need for more efficient, lightweight solutions suitable for edge deployment. Wang et al. [14] proposed an IoT and cloud-based municipal waste management solution that integrates smart containers equipped with sensors and Bluetooth connectivity and a mobile app for image-based waste classification. MobileNetV3 model was selected for the latter purpose due to its compact storage size and fast computational speed. Ekundayo et al. [15] presented a mobile-based framework for on-device inference based on quantized CNN models, without relying on cloud connectivity. Their approach targets regions with limited internet access and incorporates gamification features to promote user engagement in household recycling.

In our previous work [16], we explored the application of image classification techniques in developing an intelligent assistant for waste disposal guidance specifically tailored to Spain’s recycling framework. That study proposed and compared a series of state-of-the-art architectures suitable for this task, achieving a strong performance on the designed dataset. Additionally, we introduced a framework for combining the predictions from multiple models using aggregation functions. This strategy improved classification performance and is designed to make the system more robust when deployed in real-world environments.

Despite the promising results of our aggregation framework, deploying multiple models simultaneously presents significant practical challenges, especially for mobile or edge devices with limited computational resources, limiting the proposed system effectiveness. To address this, we extend our previous work by targeting the efficient deployment of waste classification models on IoT devices equipped with a camera sensor and low-resource environments. First, we expand our dataset with a new organic waste class and more diverse samples from various sources to improve class balance and image heterogeneity. Next, we apply model quantization techniques to reduce computational demands while maintaining acceptable accuracy. Finally, we evaluate individual quantized models and aggregated approaches to determine the most effective and efficient configuration for practical deployment. Based on these results, we also propose architectural use cases for mobile, edge, and cloud-assisted applications in real-world recycling settings.

In this paper, we address the challenge of user confusion in waste sorting, which remains a significant barrier to improving recycling outcomes despite growing efforts to promote sustainable practices. Existing solutions often lack the efficiency or portability needed for real-world deployment, especially in resource-constrained environments. To bridge this gap, we propose a lightweight, real-time waste classification framework based on quantized deep learning models and an extended, heterogeneous dataset aligned with Spain’s recycling system. Our approach explores both individual and ensemble classifiers, evaluates deployment feasibility on edge devices, and outlines architectural proposals for different IoT-enabled recycling support scenarios.

The rest of the paper is organized as follows. Section 2 describes materials and methods used for this study, including the construction of the dataset, the choice of deep learning architectures, the theoretical foundation of the model’s aggregation framework and the experimental setup. Section 3 summarizes the results obtained for the previously described experiments. Finally, a discussion on these results is presented in Section 4, laying the groundwork for future research.

## 2. Materials and Methods

In this section, we detail the methodology adopted in this study. We begin by describing the construction of the proposed dataset, highlighting its improvements over the version used in our previous work. Next, we introduce the selected deep learning architectures and justify their relevance to the task. We then present the theoretical aggregation framework employed to combine multiple model predictions. Finally, we outline the experimental setup, including training procedures and the practical setup for model aggregation.

### 2.1. Dataset Description

Building upon our previous work [16], we have developed a more comprehensive and balanced dataset for urban waste image classification. Our previous dataset combined two open collections (TrashNet [5] and TrashBox [17]) with modified class structures to align with the Spanish recycling system (https://www.ecoembes.com/en), particularly focusing on the metropolitan area of València. The original dataset comprised 17,855 images distributed across six classes. While this approach yielded promising results, we observed performance disparities between classes during model evaluation. For example, the Sigre category (medicines), with only 1016 images, exhibited a 5% gap between precision and recall compared to larger classes like Blue (4312 images). We hypothesized that class imbalance contributed to these inconsistencies.

To address this limitation and enhance robustness for real-world deployment, we have significantly expanded and rebalanced the dataset with images from multiple open collections. Additionally, a new Organic class was introduced to reflect the increasing implementation of organic waste recycling systems in many Spanish municipalities, an aspect that was missing in the original study. The new Organic category was primarily derived from the Food-101 dataset [18], which contains over 100 different types of food, including raw and cooked items. To further increase diversity and realism, we incorporated 1500 images from the Household Waste dataset [19], which include eggshells, coffee grounds, and food scraps (e.g., fruit and vegetable peels, leftovers, etc.) in realistic disposal contexts. Together, these sources provide heterogeneous examples of organic waste in both prepared and discarded states, which closely align with real-world household disposal scenarios. Although agricultural waste is not directly represented, the class was designed for consumer-level recycling assistants, where domestic food waste is the primary concern.

Since the dataset is composed of images sourced from a wide range of publicly available open datasets, the image acquisition conditions vary significantly. These include studio-quality photos with clean, uniform backgrounds, in-the-wild images of waste discarded on various surfaces (e.g., streets, grass), multi-object scenes, and images scraped from the internet. This heterogeneity is an intentional feature, promoting the generalization ability of models to different real-world visual scenarios. As we did not collect the images ourselves, detailed metadata, such as camera specifications or controlled lighting conditions, is not uniformly available. However, this diversity better reflects deployment environments where waste may appear under uncontrolled conditions. The final dataset now consists of seven classes and a total of 27.396 images, distributed as follows:

- Blue: blue container for paper and cardboard (4310 images).
- Green: green container for glass (3839 images).
- Green point: special waste (like electronic devices) requiring disposal at municipal clean point (4093 images).
- Grey: grey container for non-recyclable waste (2948 images).
- Sigre: pharmaceutical waste and expired medications for specialized SIGRE collection points at pharmacies (3833 images).
- Yellow: yellow container for plastics, packaging, and cans (4369 images).
- Organic: brown container for food scraps and organic waste (4000 images).

**Figure 1:**
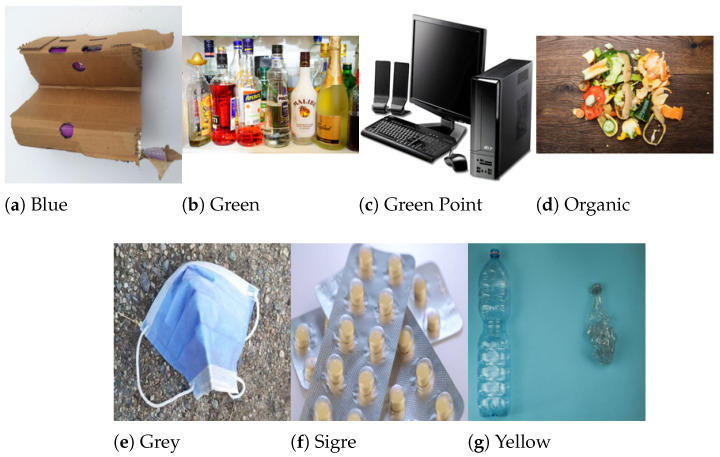
Sample images from each waste class in the final dataset.

To provide a visual reference for the dataset composition, Figure 1 displays one sample image per class. While each category encompasses a broad range of waste types and visual contexts, these examples illustrate the diversity of the dataset, which includes both studio-like images with uniform backgrounds and realistic, in-the-wild samples featuring varied lighting and environments.

A detailed mapping of original dataset sources to the established recycling classes, including constituent subcategories and number of images, is provided in Table 1. This structure maintains continuity with the previous taxonomy but introduces several key improvements. First, the dataset is more balanced, as all categories range between 3834 and 4369 images. The only exception is the Grey container class, which remains slightly smaller. This is primarily because many items considered non-recyclable in the source datasets are, under the proposed recycling scheme, assigned to specific recyclable categories. Additionally, a new category has been added for organic waste. Finally, the dataset now includes a greater diversity of images, sourced from multiple open datasets. This brings in variation in object types, lighting conditions, backgrounds, and image quality, which is expected to improve model generalization and robustness in real-world applications.

### 2.2. Network Architectures

This section provides a brief overview of the various architectures proposed for our experiments. The choice of models is primarily guided by their frequent use in related research, as illustrated in Section 1, allowing us to position our work within a broader academic context. Additionally, we aimed to include a diverse set of models—ranging from high-performance architectures to those optimized for limited-resource environments. These architectures were also chosen based on their suitability for waste classification tasks in our prior study.

**InceptionV3** [25] employs parallel multiscale feature extraction through inception modules, providing a strong balance between accuracy and efficiency. **Xception** [26] builds on Inception by replacing inception modules with depthwise separable convolutions, allowing for efficient feature extraction while maintaining high accuracy. **MobileNetV3** [27] is specifically optimized for mobile and embedded applications. It uses lightweight bottleneck blocks and inverted residuals, making it a compact yet powerful choice for edge deployment. **NASNet** [28] is generated via Neural Architecture Search and offers customizable architectures that can be adapted to diverse performance and resource requirements. **EfficientNetV2** [29] is a family of convolutional networks that apply a principled compound scaling strategy to balance depth, width, and resolution. Combined with progressive learning, this design enables strong performance and efficient resource utilization across a range of model sizes. Finally, the **YOLO** (You Only Look Once) [30] family of models, primarily developed for real-time object detection, has evolved to support a broad spectrum of computer vision tasks, including image classification, segmentation, and pose estimation. In our previous research on waste classification, YOLOv8 models outperformed almost all other architectures, including EfficientNet. This also occurs in other work in different fields, such as medicine [31], where better and faster results are also obtained with the YOLO family algorithms than with EfficientNet. Building upon this, we employed the more recent version YOLOv11 in the current study, which shows superior performance on benchmark datasets, especially on smaller variants [32]. YOLOv11 offers a range of model sizes, from ‘n’ (nano) to ‘x’ (extra-large), allowing users to select models that best fit their resource constraints and performance requirements.

### 2.3. Model Aggregation Framework

Different methods exist to combine the predictions of classification models, resulting in increased accuracy and robustness. This strategy allows for consensual decisions while mitigating the effect of outliers and individual models’ biases. Different types of aggregation functions have been proposed for this purpose. In this section, we present fundamental aggregation principles that allow the fusion of outputs from several trained classification models.

**Definition** **1** ([33,34]). *A mapping $M:{\left[0,1\right]}^{n}\to \left[0,1\right]$ is an aggregation function if it is monotone non-decreasing in each of its components and satisfies $M(\vec{0})=0$ and $M(\vec{1})=1$.*

An aggregation function *M* is an averaging or mean if

$$\min\left(x_{1},\ldots ,x_{n}\right)\leq M\left(x_{1},\ldots ,x_{n}\right)\leq \max\left(x_{1},\ldots ,x_{n}\right).$$

A relevant type of aggregation function is the Ordered Weighted Averaging (OWA) quantifier guided operators [35].

**Definition** **2.** *An OWA operator of dimension n is a mapping $\Phi :{\left[0,1\right]}^{n}\to \left[0,1\right]$ such that there exists a weighting vector $\vec{w}=\left(w_{1},\ldots ,w_{n}\right)\in {\left[0,1\right]}^{n}$ with $\sum_{i=1}^{n}w_{i}=1$, and such that*

$$\Phi \left(x_{1},\ldots ,x_{n}\right)=\sum_{i=1}^{n}w_{i}\cdot x_{\sigma (i)},$$

*where $x_{\sigma }=\left(x_{\sigma (1)},\ldots ,x_{\sigma (n)}\right)$ is a decreasing permutation on the input $\vec{x}$.*

In [35] a way to compute the weighting vector is presented:

(1) $$w_{i}=Q\left(\frac{i}{n}\right)-Q\left(\frac{i-1}{n}\right),$$

where *Q* is a fuzzy linguistic quantifier, such as, for instance,

(2) $$Q\left(r\right)=\;\left\{\begin{array}{cc} 0 & \text{if}\;0\leq r<a,\\ \frac{r-a}{b-a} & \text{if}\;a\leq r\leq b,\\ 1 & \text{if}\;b<r\leq 1, \end{array}\right.$$

with a,b,r∈[0,1].

**Figure 2:**
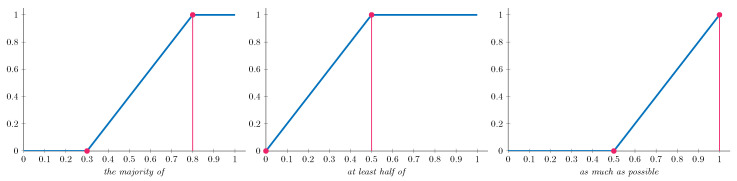
Fuzzy linguistic quantifiers used in the experiments. Each plot represents a fuzzy membership function over the [0, 1] interval, associated with a linguistic quantifier. These functions model the degree to which a given proportion satisfies the meaning of the corresponding quantifier. Pink dots represent the construction parameters for each quantifier.

For our experiments, we implement three specific OWA operators, as detailed in Table 2, each representing a different linguistic label with corresponding construction parameters. Figure 2 visually represents these linguistic quantifiers. These graphical representations illustrate how each quantifier behaves across the input domain, helping to visualize how the different aggregation strategies emphasize specific prediction patterns when combining outputs from multiple models.

**Definition** **3.** *A function $m:2^{N}\to \left[0,1\right]$ is a fuzzy measure if, for all X,Y⊆N, it satisfies the following properties:*

*1.* * * *Increasingness: if X⊆Y, then m(X)≤m(Y);*
*2.* * * *Boundary conditions: m(∅)=0 and m(N)=1.*

An example of a commonly used fuzzy measure is the power measure, which we use in this work:

(3) $$m_{q}\left(X\right)={\left(\frac{\left|X\right|}{n}\right)}^{q},\;with\;q>0,$$

where |X| is the number of elements to be aggregated, *n* the total number of elements and q>0. We have selected this measure due to the performance obtained in terms of accuracy in classification problems [36,37].

**Definition** **4** ([38]). *The discrete Choquet integral, related with the fuzzy measure m, is the function $C_{m}:{\left[0,1\right]}^{n}\to \left[0,1\right]$, defined, for all $\vec{x}\in {\left[0,1\right]}^{n}$, by*

(4) $$C_{m}\left(\vec{x}\right)=\sum_{i=1}^{n}\left(x_{\left(i\right)}-x_{\left(i-1\right)}\right)\cdot m\left(A_{\left(i\right)}\right),$$

*where $\left(x_{\left(1\right)},\ldots ,x_{\left(n\right)}\right)$ is an increasing permutation of $\vec{x}$, $x_{\left(0\right)}=0$ and $A_{\left(i\right)}=\left\{\left(i\right),\ldots ,\left(n\right)\right\}$ is the subset of indices of n−i+1 largest components of $\vec{x}$.*

**Definition** **5** ([39]). *The discrete Sugeno integral is defined concerning a fuzzy measure m by:*

(5) $$Su_{m}\left(\vec{x}\right)=\overset{n}{\underset{i=1}{⋁}}\left(x_{\left(i\right)}\wedge m\left(A_{\left(i\right)}\right)\right)$$

*where (i) is a permutation such that $x_{\left(i-1\right)}\leq x_{\left(i\right)}$ for all i=1,…,n, with $x_{\left(0\right)}=0$ and $A_{\left(i\right)}=\left\{\left(1\right),\ldots ,\left(i\right)\right\}$.*

### 2.4. Experimental Configuration

The dataset outlined in Section 2.1 was partitioned into three distinct subsets: 70% allocated for training, 20% reserved for validation, and the remaining 10% designated for testing. All network models discussed in Section 2.2 were trained on the training subset using an NVIDIA A40 GPU with 48 GB of memory (NVIDIA Corporation, Santa Clara, CA, USA). To enhance model generalization, several data augmentation methods were applied randomly during training, including shear transformations (range = 0.2), zoom operations (range = 0.2), and horizontal flips. All input images were resized to a uniform resolution of 224×224 pixels to ensure compatibility across architectures.

Findings of our previous study [16] guided the selection of optimal training configurations, showing that ImageNet-based transfer learning achieved significantly superior results compared to training from scratch, and informing hyperparameter settings. Thus, due to their peculiarities, YOLO models were trained for 100 epochs with a 0.01 initial learning rate. In comparison, all remaining models were trained for eight epochs using the TensorFlow (v2.12.1) framework and an initial 0.001 learning rate. Model weights reaching the highest accuracy on the validation set were selected for performance evaluation on the test set and subsequent experiments.

Following the training phase, all models underwent post-training quantization to reduce their computational footprint and enable deployment on resource-constrained devices. Specifically, weights and activations were converted from floating-point representations to 8-bit integers using TensorFlow Lite’s (v2.12.1) full integer quantization scheme. This process significantly decreases model size and inference time while aiming to preserve predictive performance. For YOLO-based models (Ultralytics v8.3.89), the trained PyTorch (v2.5.0) checkpoint was exported to ONNX (v1.17.0) and statically quantized to 8-bit weights and activations (per-channel QDQ) using ONNX Runtime (v1.21.0) with representative data calibration. Quantized versions of the models were then evaluated using the same test set to assess the trade-off between efficiency and accuracy. Quantized models were evaluated on a CPU-only environment using an Intel(R) Xeon(R) E5-2680 v4 @ 2.40 GHz processor (Intel Corporation, Santa Clara, CA, USA), without GPU acceleration, to simulate deployment on resource-constrained systems.

In our previous work [16], we proposed a setup in which all trained models independently predicted class probabilities for each input image, and these outputs were subsequently combined using the aggregation functions described in Section 2.3. While this approach showed promising improvements in classification performance, it also introduced significant computational overhead due to the simultaneous use of multiple models. To address this limitation, in the present study, we apply the aggregation framework exclusively to quantized models, aiming to retain ensemble benefits while improving efficiency. We conducted a model selection procedure in which aggregation accuracy was first computed using all quantized models. Then, by iteratively removing one model at a time and evaluating the resulting performance, we progressively reduced the ensemble. At each step, the model whose exclusion yielded the highest accuracy was removed. This process was repeated until a minimal subset remained. As a result, for each aggregation function, we obtain a performance curve as a function of the number of aggregated models, helping to identify the most efficient trade-off between accuracy and computational cost.

To further assess the deployability of the proposed individual quantized models in real-world conditions, we conducted two additional experiments. First, a representative subset of quantized models was evaluated on a Raspberry Pi 5 (Raspberry Pi Ltd., Cambridge, UK) to measure inference time and classification performance in a constrained hardware environment. This single-board device features a quad-core ARM Cortex-A76 processor (Broadcom Inc., San Jose, CA, USA), making it ideal for local processing tasks and edge AI applications. The selected models include YOLOv11n, MobileNetV3Small, InceptionV3, and EfficientNetV2-B0. These architectures were chosen to reflect a diverse spectrum of the accuracy–efficiency trade-off observed in earlier experiments.

**Figure 3:**
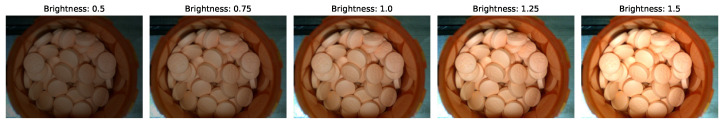
Effects of varying brightness factors (from 0.5 to 1.5) on a sample waste image used to simulate lighting condition changes for robustness testing.

Second, we conducted a robustness evaluation simulating variable lighting conditions. We selected the quantized YOLOv11n model due to its low resource usage and strong accuracy–efficiency balance across all tested configurations. Test images were synthetically augmented using varying brightness factors (see Figure 3), and classification accuracy was recorded as a function of brightness. This setup approximates real-world environmental variability, such as shadows or strong light exposure, and provides a preliminary assessment of model stability under common deployment challenges.

## 3. Results

This section reports the results of the experiments proposed in Section 2.4. To assess the classification performance, a confusion matrix is built, from which the number of True Positives (TP), True Negatives (TN), False Positives (FP), and False Negatives (FN) is computed for each class. These values are then used to calculate commonly used evaluation metrics: precision (PREC), recall (REC), and the $F_{1}$ score, defined as follows:

(6) $$\mathrm{PREC}=\frac{\mathrm{TP}}{\mathrm{TP}+\mathrm{FP}},\;\mathrm{REC}=\frac{\mathrm{TP}}{\mathrm{TP}+\mathrm{FN}},\;F_{1}=\frac{2\cdot \mathrm{PREC}\cdot \mathrm{REC}}{\mathrm{PREC}+\mathrm{REC}}$$

Table 3 compares these performance metrics of both quantized and non-quantized models over the test dataset. These metrics are computed per class and then macro-averaged, meaning the average is taken across all classes with equal weight, regardless of class frequency. Resource-related metrics such as model size and average inference time are also contrasted. Table 4 further analyzes the impact of quantization by presenting the variation in key metrics between original and quantized models. It includes both absolute and relative changes in classification accuracy, inference time, and model size, allowing for a detailed assessment of quantization effects. Particularly, the final column, Acc/MB (Quantized), reflects the accuracy-to-size efficiency of each quantized model and serves as a practical indicator of performance per storage unit.

Non-quantized YOLOv11x exhibited the highest performance across all evaluated metrics, achieving an $F_{1}$ score of 94.8%. Model quantization, in general, led to a substantial reduction in model size and inference time, while only marginally affecting classification performance. For instance, the quantized YOLOv11n model, which is the second fastest with an inference time of just 21 ms (equivalent to 47.6 frames per second) and a model size of only 1.7 MB, achieves a commendable accuracy of 92.2%, representing merely a 0.5% drop compared to its full-precision version. However, models specifically designed for deployment in resource-constrained environments (such as NasNet-Mobile and MobileNetV3) experienced significant performance degradation upon quantization. This behavior is consistent with prior findings [40,41], which attribute the performance degradation on depthwise separable convolution-based models to components such as Batch Normalization and ReLU6 activations inserted between depthwise and pointwise layers, which are highly sensitive to quantization errors. Although several strategies have been proposed to address this issue, such as quantization-aware training or modifying core layer designs, we focused on post-training static quantization due to deployment simplicity. Exploring these optimization techniques for specific models is out of the scope of this work and constitutes a promising direction for future research. It is worth noting that, excluding these lightweight architectures which experienced substantial performance degradation, the Acc/MB ratio appears to be a reliable indicator for selecting models that offer a favorable trade-off between classification performance and resource efficiency. Finally, while quantized models such as Xception and EfficientNetV2-S demonstrated strong classification performance and significant reductions in model size, their inference times did not improve as expected. In our CPU-based evaluation setup, these models exhibited longer inference times than their full-precision counterparts. This behavior can be attributed to the limited hardware acceleration for quantized operations on the tested CPU and potential overhead introduced by the inference framework when handling int8 computations.

**Figure 4:**
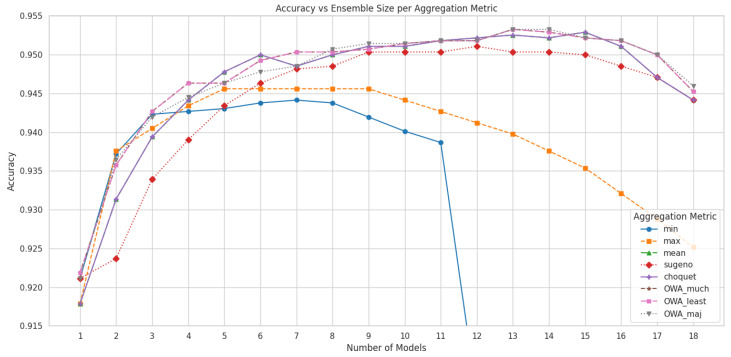
Accuracy of aggregated predictions depending on the number of models, for each aggregation function.

The impact of model aggregation on classification performance is illustrated in Figure 4, which shows the variation in test accuracy as a function of the number of quantized models used in each aggregation strategy. Each curve corresponds to a different aggregation function, highlighting how performance evolves as models are incrementally added or removed from the ensemble. Based on this analysis, Table 5 presents the final performance metrics—accuracy, precision, recall, and $F_{1}$ score—for each aggregation method using the optimal number of models. Notably, most aggregation strategies outperformed all individual models, with OWA operators achieving the strongest performance, indicating that combining predictions from multiple quantized classifiers effectively enhances classification robustness. The only exception to this trend was the YOLOv11x-cls model, whose performance exceeded the weakest-performing aggregation methods, namely the min and max operators.

**Figure 5:**
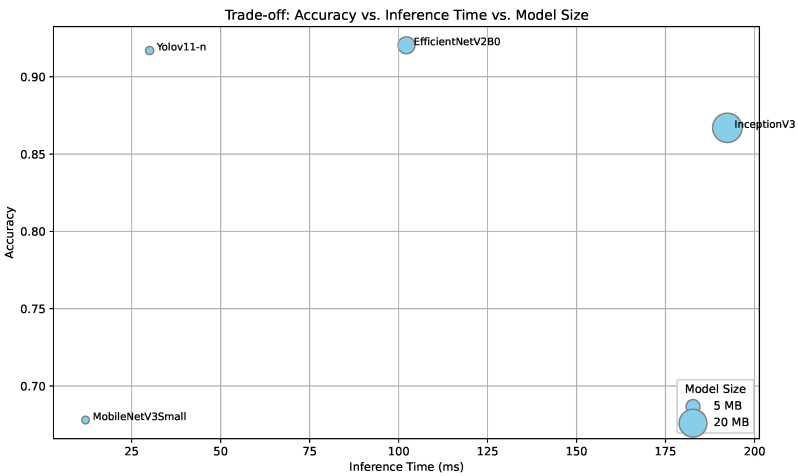
Trade-off between classification accuracy, inference time (in milliseconds), and model size (represented by point size) for selected quantized models evaluated on a Raspberry Pi.

Figure 5 presents the trade-off between classification accuracy, inference time, and model size for a representative set of quantized models evaluated on a Raspberry Pi. EfficientNetV2B0 achieved the highest accuracy (92.04%) with an inference time just over 100 ms, making it a strong candidate for near real-time applications where latency constraints are moderate. In contrast, the quantized YOLOv11-n model reached a slightly lower accuracy (91.7%) but offered significantly faster inference (30.05 ms) and a more compact storage size, making it the most suitable option for real-time deployment in highly resource-constrained environments. This confirms the practical viability of our proposed solution and offers two strong deployment paths depending on application requirements.

As expected, all models exhibited increased inference times on the Raspberry Pi compared to CPU measurements. However, the relative slowdown was more pronounced in larger architectures. For instance, EfficientNetV2-B0 and InceptionV3 experienced latency increases of more than a factor of 2 and 3, respectively, whereas lightweight models like MobileNetV3 Small and YOLOv11-n showed only modest increases (e.g., +2.4 ms and +9 ms). This suggests that more compact models tend to scale better across constrained hardware, reinforcing their suitability for edge deployment.

**Figure 6:**
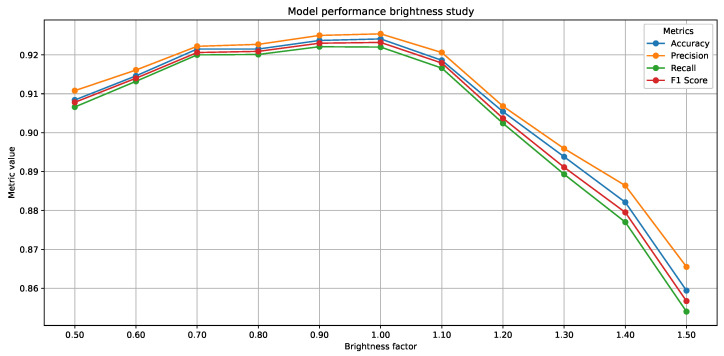
Brightness study on quantized YOLOv11n-cls on the test dataset to assess its robustness under changing lightning conditions.

The results of the robustness tests under varying lightning conditions (see Figure 6) show that the model’s performance remains relatively stable across a broad brightness spectrum, with behavior that reflects its ability to adapt to different lighting conditions. According to the data obtained, key metrics such as Precision, Recall, and F1-Score range from 0.91 to 0.92 over a brightness range of 0.7 to 1.0, indicating remarkable consistency in normal to slightly dark lighting conditions. However, the model proves to be more robust against dark than over-illumination conditions; for example, at a brightness factor of 0.6, the metrics barely drop below 0.90, whereas at a factor of 1.4, the F1-Score drops to approximately 0.86 and the recall to 0.87, reflecting a greater sensitivity to overexposure. While these findings confirm the model’s robustness under most realistic lighting conditions, deployments in environments with strong lighting or glare may benefit from preprocessing techniques such as dynamic exposure adjustment or hardware-level lighting control. Further real-world testing under diverse environmental conditions is planned for future work.

## 4. Discussion

This work presents a comprehensive evaluation of quantized deep learning models and aggregation strategies for automated waste classification, with a focus on compatibility with the Spanish recycling framework and model efficiency. A major contribution is the development of a new, diverse, and well-balanced dataset that expands previous efforts by including an organic waste class and enhancing image heterogeneity. This facilitates more realistic training and evaluation, ensuring better generalization in practical applications.

Experimental results show that quantized models can retain high classification performance while significantly reducing model size and inference time. YOLO-based classifiers achieved the strongest results, with YOLOv11x delivering the highest overall accuracy and $F_{1}$ score. However, this came at the expense of increased computational complexity. In contrast, the quantized YOLOv11n model demonstrated a strong balance between efficiency and accuracy—achieving 92.2% accuracy with an inference time of 21 ms and a model size of just 1.7 MB—making it an excellent candidate for deployment in edge environments.

Our aggregation framework, extended from previous work, enabled a systematic exploration of ensemble strategies using only quantized models. While ensembles generally outperformed individual classifiers, except for YOLOv11x, the gains were modest (e.g., an improvement of approximately 0.5%). This raises questions about whether the marginal accuracy boost justifies the increased computational cost, especially when model simplicity and efficiency are critical. Nonetheless, this work advances the feasibility of deploying ensembles by reducing their computational footprint. By quantizing all constituent models and optimizing ensemble size, we showed that strong performance can be maintained with a smaller subset of classifiers.

### 4.1. Architectural Deployment

To address different operational scenarios, we propose diverse deployment strategies customized to the available computational and connectivity resources. In low-connectivity environments, such as rural areas or remote zones, real-time classification using lightweight, quantized models on embedded IoT devices is a promising direction. Our results show that models like quantized YOLOv11n provide an excellent trade-off between accuracy, size, and speed, making them well-suited for such edge applications.

A representative hardware prototype is the low-cost smart device, developed by our team, for real-time plant disease detection [42]. This system, known as Plantillo, is built around a Raspberry Pi Zero W (Raspberry Pi Ltd., Cambridge, United Kingdom) and integrates a camera, a deep learning classification model, a local display interface, and Wi-Fi connectivity. The system captures data through the Raspberry Pi Camera Module 3, which features a 12MP Sony IMX708 sensor (Sony Corporation, Tokyo, Japan) with HDR and improved low-light sensitivity—making it a reliable and compact visual sensing component for edge deployment scenarios. The original device performs real-time on-device inference using a quantized MobileNetV2 model without requiring an Internet connection, and can optionally transmit metadata to the cloud for storage or analysis. Thanks to its compact size and low power requirements, it can be mounted on drones, robots, or stationary units.

The modularity and adaptability of Plantillo demonstrate the feasibility of deploying deep learning models for waste classification under similar constraints. A comparable device could be integrated into smart bins for automatic waste classification. That solution would reduce infrastructure dependencies while supporting the development of sustainable waste management practices, particularly in under-resourced or rural regions.

In this context, we do not recommend aggregated models for highly constrained edge devices. Instead, efficient quantized models such as YOLOv11n are better suited for low-power systems. However, ensemble approaches remain relevant for cloud-enabled applications with sufficient resources, where maximizing classification reliability may be prioritized. For instance, in user-facing mobile applications, even infrequent misclassifications can negatively impact user trust. Therefore, marginal improvements in average accuracy can still be valuable in such settings.

To address the ensemble’s computational limitations, future work should explore model distillation [43], a technique in which a compact student model learns to reproduce the behavior of a larger model or ensemble. In our case, a lightweight classifier could be trained to approximate the soft probability distributions produced by the ensemble. This offers a promising strategy for reducing inference cost while preserving most of the benefits of aggregation.

### 4.2. Relevance for IoT-Based Smart Services

Our findings align with broader efforts to enable IoT-based smart city and smart rural services. In urban environments, camera-equipped waste collection systems or public recycling stations could benefit from real-time classification models running on the edge or connected via 5G and cloud services. In rural areas, self-contained, low-cost devices with onboard waste recognition capabilities can help promote recycling behavior where infrastructure is sparse. In both settings, the combination of deep learning and efficient sensor-based processing supports scalable, responsive waste management services, key pillars in sustainable urban and environmental planning.

### 4.3. Future Research Directions

While this study evaluated quantized models on a CPU and a Raspberry Pi, additional testing on hardware specifically optimized for deep learning at the edge seems a promising direction. Future research should investigate deployment on accelerators such as Google’s Coral TPU or NVIDIA Jetson Nano, which are increasingly used in embedded AI applications and offer dedicated inference capabilities for convolutional models.

Another important step is to assess the system’s behavior in real-world environments. This includes integrating the model into a smart bin or similar operational context to evaluate its robustness under dynamic conditions such as varying lighting, occlusion, and background clutter. These field trials can reveal practical limitations not evident in controlled experiments and inform improvements in model design or preprocessing strategies.

Additionally, while ensemble-based aggregation improves classification reliability, it comes with increased computational demands. Future work should explore model distillation, a technique where a compact student model is trained to mimic the soft outputs of a more complex ensemble. This strategy has the potential to retain much of the ensemble’s performance while drastically reducing inference cost, making it a promising solution for resource-constrained deployments.

Finally, extending the system to support real-time detection, as well as integrating it with larger sensor networks and urban data systems, represents an important next step toward operational deployment.

## Figures and Tables

**Table 1:** Summary of all dataset subcategories grouped by proposed recycling classification, including original and newly incorporated sources and image counts.

Final Class	Subcategories	Image Source	# Images
Blue	Paper, cardboard	TrashNet [5]	997
	Cardboard, paper cups, paper, news paper	TrashBox [17]	3315
Green	Glass	TrashNet	501
	Glass	TrashBox	2027
	Glass bottles, food jars, cosmetic flasks	Household Waste [19]	1312
Green point	Metal	TrashNet	410
	Electrical cables, electronic chips, laptops, small appliances, smartphones, construction scrap, metal containers, other metal objects	TrashBox	3683
Grey	Trash	TrashNet	137
	Gloves, masks, cigarette butt, plastic containers, plastic cups	TrashBox	2887
	Diapers, masks, napkins, cigarettes	Garbage Dataset V2 [20]	411
Sigre	Medicines, syringes	TrashBox	1016
	Blisters	Medicine pack dataset [21]	437
	Blisters	Ultralytics medical pills [22]	23
	Pills in bottles	UMich [23] (NLM20)	557
	Synthetic pills	Synthetic pills [24]	1800
Yellow	Plastic	TrashNet	482
	Beverage cans, spray cans, tetra pak, plastic bags, plastic bottles	TrashBox	2887
	Plastic cutlery, straws, aluminum cans	Household Waste	1000
Organic	Eggshells, coffee grounds, food waste	Household Waste	1500
	Food101 (misc. food, randomly selected)	Food101 [18] (train set)	2500

**Table 2:** OWA operators, representing linguistic labels and their construction parameters.

#	*a*	*b*	Linguistic Label
${\mathrm{OWA}}^{1}$	0.3	0.8	*the majority of*
${\mathrm{OWA}}^{2}$	0	0.5	*at least half of*
${\mathrm{OWA}}^{3}$	0.5	1	*as much as possible*

**Table 3:** Comparison of performance and efficiency metrics between original and quantized models. Boldface highlights the best values in each metric.

Model	Quantized	Acc	PREC	REC	$F_{1}$	Inf. Time (ms)	Model Size (MB)
MobileNetV3-Small	✗	88.3	88.7	88.1	88.3	52.3	7.3
	✓	68.1	73.9	66.9	67.0	**9.6**	**1.5**
MobileNetV3-Large	✗	92.2	92.4	92.0	92.2	50.7	17.5
	✓	56.2	70.6	55.8	54.1	33.8	3.8
NasNet-Mobile	✗	87.8	88.3	87.5	87.8	71.5	23.8
	✓	20.2	27.5	19.8	14.5	58.6	5.6
NasNet-Large	✗	89.2	89.4	89.0	89.2	168.5	349.3
	✓	89.2	89.4	89.0	89.1	372.5	87.5
InceptionV3	✗	87.1	87.3	87.0	87.1	70.3	95.7
	✓	87.0	87.1	86.9	87.0	57.0	22.3
Xception	✗	87.9	88.4	87.8	87.9	84.8	91.9
	✓	88.1	88.5	87.9	88.0	99.0	22.1
EfficientNetV2-B0	✗	92.4	92.6	92.3	92.4	62.3	30.6
	✓	91.8	92.0	91.7	91.8	46.8	7.4
EfficientNetV2-B1	✗	92.8	93.0	92.6	92.8	64.7	34.5
	✓	92.2	92.4	91.9	92.1	61.4	8.6
EfficientNetV2-B2	✗	92.7	93.0	92.6	92.8	68.2	42.3
	✓	92.1	92.4	91.9	92.1	69.2	10.6
EfficientNetV2-B3	✗	92.4	92.6	92.3	92.4	75.1	59.0
	✓	91.9	92.1	91.8	91.9	91.8	15.1
EfficientNetV2-L	✗	90.8	90.6	90.7	90.7	203.6	458.3
	✓	85.7	86.1	85.7	85.7	561.3	122.3
EfficientNetV2-M	✗	90.7	90.7	90.7	90.7	124.2	211.4
	✓	89.2	89.1	89.2	89.1	268.9	56.7
EfficientNetV2-S	✗	92.3	92.3	92.2	92.2	94.2	85.9
	✓	91.7	91.7	91.6	91.6	145.6	22.9
YOLOv11n-cls	✗	92.7	92.9	92.6	92.3	38.6	3.1
	✓	92.2	92.4	92.0	91.8	21.0	1.7
YOLOv11s-cls	✗	93.7	93.7	92.1	93.7	101.3	10.5
	✓	93.0	93.5	91.7	93.0	65.8	5.5
YOLOv11m-cls	✗	93.9	93.8	93.8	93.8	251.3	19.9
	✓	92.4	92.7	92.2	92.4	187.8	10.3
YOLOv11l-cls	✗	93.9	93.9	93.8	93.8	330.9	24.7
	✓	93.7	93.8	93.7	93.8	234.3	12.8
YOLOv11x-cls	✗	**94.8**	**94.8**	**94.8**	**94.8**	587.0	54.4
	✓	94.5	94.6	94.5	94.2	477.9	27.7

**Table 4:** Variation, in absolute and percentage terms, in key metrics between original and quantized models, ordered by accuracy per MB after quantization. All differences are computed as *quantized* minus *original*. Negative values in absolute accuracy, time and size indicate a drop in performance or an improvement in inference time and model size, respectively. Acc/MB reflects classification accuracy normalized by model size, providing an indicator of efficiency.

Model	ΔAcc (%)	%Acc Loss	ΔTime (ms)	%Time Gain	ΔSize (MB)	%Size Reduction	Acc/MB (Quant)
YOLOv11n-cls	−0.5	0.54	−17.6	45.60	−1.4	45.16	**54.235**
MobileNetV3-Small	−20.2	22.88	−42.7	81.64	−5.8	79.45	45.400
YOLOv11s-cls	−0.7	0.75	−35.5	35.04	−5.0	47.62	16.909
MobileNetV3-Large	−36.0	39.05	−16.9	33.33	−13.7	78.29	14.789
EfficientNetV2-B0	−0.6	0.65	−15.5	24.88	−23.2	75.82	12.405
EfficientNetV2-B1	−0.6	0.65	−3.3	5.10	−25.9	75.07	10.721
YOLOv11m-cls	−1.5	1.60	−63.5	25.27	−9.6	48.24	8.971
EfficientNetV2-B2	−0.6	0.65	1.0	−1.47	−31.7	74.94	8.689
YOLOv11l-cls	−0.2	0.21	−96.6	29.19	−11.9	48.18	7.320
EfficientNetV2-B3	−0.5	0.54	16.7	−22.24	−43.9	74.41	6.086
EfficientNetV2-S	−0.6	0.65	51.4	−54.57	−63.0	73.34	4.004
Xception	0.2	−0.23	14.2	−16.75	−69.8	75.95	3.986
InceptionV3	−0.1	0.12	−13.3	18.92	−73.4	76.70	3.901
NasNet-Mobile	−67.6	76.99	−12.9	18.04	−18.2	76.47	3.607
YOLOv11x-cls	−0.3	0.32	−109.1	18.59	−26.7	49.08	3.412
EfficientNetV2-M	−1.5	1.65	144.7	−116.51	−154.7	73.18	1.573
NasNet-Large	0.0	0.00	204.0	−121.07	−261.8	74.95	1.019
EfficientNetV2-L	−5.1	5.62	357.7	−175.69	−336.0	73.31	0.701

**Table 5:** Comparison of aggregated model test performance (in %). The maximum values for Acc, PREC, REC, and $F_{1}$ are shown in bold. The Model Count column indicates the number of individual models aggregated in each case, selected as the combination that achieved the best performance for each aggregation function.

#	Model Count	Acc	PREC	REC	$F_{1}$
min	7	94.41	94.46	94.41	94.45
max	5	94.56	94.60	94.56	94.56
mean	15	95.29	95.35	95.29	95.37
Choquet	15	95.29	95.35	95.29	95.37
Sugeno	12	95.11	95.16	95.11	95.15
${\mathrm{OWA}}^{1}$	13	**95.33**	**95.39**	**95.33**	**95.38**
${\mathrm{OWA}}^{2}$	13	**95.33**	**95.39**	**95.33**	**95.38**
${\mathrm{OWA}}^{3}$	13	**95.33**	**95.39**	**95.33**	**95.38**

## Data Availability

The datasets used in the study are openly available. The composition of the final dataset is detailed in Section 2.1.
